# Generative AI for Establishing the Chief Complaint

**DOI:** 10.3390/jcm15207912

**Published:** 2026-10-13

**Authors:** Vera Sorin, Eyal Klang

**Affiliations:** 1 Department of Radiology, Mayo Clinic, Rochester, MN 55905, USA; 2 Department of Radiology, Beth Israel Deaconess Medical Center, Harvard Medical School, Boston, MA 02215, USA; 3 BRIDGE GenAI Lab, Beth Israel Deaconess Medical Center, Boston, MA 02215, USA

**Keywords:** chief complaint, generative artificial intelligence, patient intake, triage, history-taking, clinical assessment

## Abstract

Establishing the chief complaint requires identifying the patient’s main symptoms or concerns. This information is essential for triage and subsequent clinical assessment. However, patients may struggle to describe their symptoms or leave important concerns unspoken. Also, clinicians have limited time to ask further questions during intake. Generative AI could help by asking patients what is troubling them, when their symptoms began, and what other concerns they have. It could prepare a short summary for patients to correct and clinicians to review. Clinical studies of AI-assisted intake have reported shorter physician consultations and more complete recorded histories. These findings do not show whether AI helps clinicians establish the chief complaint more accurately. We propose an evaluation framework centered on the proportion of patients whose main symptoms or concerns are missing or incorrectly described after AI-assisted intake. Studies should compare what patients wanted to discuss with what the AI agent asked about and included in its summary. They should also examine whether concerns requiring urgent assessment were missed. The clinical goal is a clear description of the patient’s main symptoms and concerns that helps clinicians judge urgency and decide what needs attention.

## 1. Introduction: Establishing the Chief Complaint

The chief complaint identifies the patient’s main symptoms or concerns and helps the clinical team decide what needs assessment. During triage, the chief complaint helps staff judge urgency alongside their observations and the patient’s vital signs [1,2]. During the consultation, it helps the clinician decide which questions to ask and what to examine. The symptoms may be clear before their cause is known.

Patients do not always describe their main symptoms or concerns in their first few words. A patient may ask for a test without explaining what prompted the request. A patient who attributes symptoms to a medication may explain the suspected cause without describing the symptoms themselves. Even a familiar symptom word may need further questions. Someone who reports feeling dizzy may be describing spinning, unsteadiness, or feeling faint [3]. Asking what the sensation feels like helps establish what they are experiencing. The clinician also needs to understand when it began and what has changed.

The main concern need not be a new physical symptom. A patient seeking help after reading an abnormal test result may have no new symptoms to report. The clinician needs to know which result prompted the concern and what the patient wants explained [4]. Intake should let patients report a concern about a test result without selecting a physical symptom.

Additional concerns may take longer to emerge. Interviews with 40 patients with complex health needs and 17 primary care physicians identified short visits, differing priorities, and embarrassment as obstacles to discussing patients’ concerns [5]. A patient may begin with the problem that is easiest to describe and mention another only when invited. A brief intake conversation may end before that invitation [6,7]. The resulting chief complaint can accurately describe the first symptom while leaving out the concern that matters most to the patient or needs earlier assessment [8].

How clinicians ask can affect what patients report [9]. In an analysis of 302 consultations, general opening questions were associated with longer patient descriptions and more distinct symptoms than questions seeking confirmation of a problem already named [10]. A study involving 224 patients and 20 family physicians examined how clinicians invited additional concerns. After baseline visits, physicians were assigned to ask a question using either “something else” or “anything else.” When physicians used “something else,” fewer concerns went unaddressed than during baseline visits, without an increase in visit length [11].

Patient and physician descriptions of the chief complaint can also differ. In a study of 6722 emergency department patients, the descriptions disagreed in 32.6% of cases; patients reported a median of two symptoms and physicians one [12]. Disagreement does not establish which description was more appropriate. A clinician may need to assess a more urgent problem before addressing the patient’s main concern. However, that clinical priority does not remove the patient’s other concerns. A useful intake record should retain both the symptoms requiring prompt review and the concern the patient hoped to address.

A clearly described, isolated symptom may need only a few questions about timing and severity. A vague description or several concerns may require more discussion. The purpose is to obtain the information needed for the next clinical decision, rather than complete every part of the medical history. Information about an urgent problem must reach the clinical team before a long interview is finished. The chief complaint may also need updating when the patient’s symptoms change after booking or while waiting for assessment.

Generative AI could ask patients these questions during intake and prepare a summary for the clinical team. Conversational systems have been tested in simulated consultations and in history-taking with patients [13,14]. We propose a framework for evaluating chief-complaint accuracy by comparing patients’ concerns with the AI conversation, summary, and subsequent clinical assessment.

## 2. Generative AI During Intake

An AI-assisted intake conversation could begin by asking what is troubling the patient. The patient would not have to choose a symptom category before describing the problem. The system could use that description to select a follow-up question, ask about details that remain unclear, and summarize the answers. The AI agent could ask whether the patient has a different or additional concern from the one recorded at booking.

The follow-up questions should help the patient explain the symptom in ordinary language. Voice input or the patient’s preferred language could help some patients describe symptoms, while others would need help or a conversation with staff. Asking one question at a time would make it easier to see which question an answer addresses. An unclear response could lead to a simpler question or a request for an example. For a patient who describes tiredness, asking what has become difficult during the day may provide more useful information than repeatedly asking for a severity score. A patient who cannot remember when symptoms began should be able to give an approximate date or duration without being pressed to supply an exact date. The system should keep track of questions it has already asked so patients do not have to repeat an answer. Further questions should address something still unclear about the main symptoms or concerns, rather than simply lengthen the history (Table 1).

For tiredness, the AI agent could ask, “What makes it difficult to finish an activity you would normally manage?” The answer might concern difficulty staying awake, lack of strength, or stopping to catch a breath. Asking about a recent occasion gives the patient a way to explain what happened. A report of falling asleep during the day could lead to questions about sleepiness and disrupted sleep. Someone who stops because of breathing difficulty could be asked when that began and whether it also happens at rest. The patient may describe more than one problem, and the questions should allow for that. An answer such as “I just cannot do as much” would need further clarification rather than an automatic change from tiredness to weakness. The AI agent could ask which activity has become difficult and what prevents the patient from completing it. The chief complaint could then describe the symptom and its effect on activity using details the patient actually provided.

The conversation could also distinguish the start of a symptom from a recent change. Consider a hypothetical patient who asks for thyroid testing. Further questions establish tiredness for several months and poor sleep after a shift change three weeks ago. The patient then corrects a summary that places the onset of tiredness at the shift change. The corrected summary could read: “Tiredness for several months; poor sleep after a shift change three weeks ago; requests thyroid testing.” These details give the clinician specific information to assess without asserting that thyroid disease or the shift change caused the symptoms.

The AI agent should ask patients to explain conflicting answers. A patient who mentions both last week and this morning may be describing the onset and worsening of a symptom. The system could ask what happened at each time. The patient might instead explain that the symptom stopped and returned, or that the first answer was mistaken. That answer should determine the timeline recorded in the summary. When a caregiver answers, the summary should identify who reported each symptom or observed change.

Patients could give and check their answers by voice as well as text. The PreA chatbot collected histories from patients or designated caregivers using either form of input [14]. A proposed intake system could show a spoken answer as text and let the patient correct words it recorded incorrectly. It could also read a question aloud or rephrase it when asked, without restarting the interview. Patient review could use the same options. Someone who struggles to edit a written summary could explain a correction aloud, then hear the revised sentence. Patients should be able to check a summary in the language they used, even when the clinician receives a translation. Caregivers could add observations without replacing the patient’s answers. A family member might describe a change in walking that the patient has not noticed. The AI agent could ask who observed the change and let the patient add their own description before sending the summary.

Before asking many detailed questions about one symptom, the system could ask what else is troubling the patient. It could then ask which concern the patient most wants addressed [15]. Patients should also be able to say that they would prefer to discuss a concern directly with a clinician. The summary could note that the patient has another concern to discuss directly with the clinician.

An additional concern may change how urgently the patient needs assessment. In a hypothetical example, a patient who initially describes a rash then mentions breathlessness that began that morning. The system could display both complaints and bring the new breathing symptom to the triage clinician’s attention before continuing to ask about the rash. The clinician could then decide whether prompt assessment is needed. Both complaints should remain visible during the assessment.

The summary should put the main symptoms and concerns where the receiving clinician can find them quickly [17]. A short chief-complaint statement could include relevant timing and recent changes, followed by additional concerns. Longer background information could remain available separately. The summary should retain the patient’s own words when a more specific medical term would change their meaning. A suspected medication side effect should be recorded as the patient’s concern, not a confirmed cause.

Existing clinical information could reduce repeated questioning. A patient should not have to type a complete medication list to explain a new symptom when the service already has that list. The system could ask the patient to confirm relevant changes. However, previous symptoms should not be treated as current without confirmation. The receiving clinician should be able to tell which information came from the current conversation and which came from earlier records [18].

Patients could review the summary and correct individual details [16]. A change to timing or the description of a symptom should appear in the version the clinician receives. The summary should identify unanswered questions. The summary should not record a symptom as denied unless the patient actually denied it. Clinicians would also need a way to check an unclear phrase against the relevant patient response without reading the entire conversation.

Patients should be told who can read the intake conversation, how long recordings and transcripts are stored, and whether the information may be reused. Services should restrict access to authorized staff, store the data securely, and obtain separate patient consent before reusing identifiable information for model training.

For triage, the information needs to be available as it is obtained. Waiting until the interview ends to report a potentially urgent concern could delay assessment. The clinical service would need to specify which responses require staff review, who receives them, and how that person is notified. Patients must still be able to reach staff directly. Patients must be able to receive urgent attention without finishing the chatbot interview or approving its summary. The summary contributes symptom information; a triage clinician must also assess the patient [1].

Patients should be able to report changes while they wait. A patient could add that a symptom has worsened or that another concern was omitted. The updated information should reach the staff responsible for reassessment, rather than remain in a separate chat history.

The clinician could then use the updated summary during the consultation [19]. They could confirm the chief complaint, ask about missing details, and examine the patient. The patient should not have to repeat the whole interview solely because the AI output is unavailable or difficult to read. However, a new question remains appropriate when the answer is uncertain or the patient’s condition has changed.

**Figure 1:**
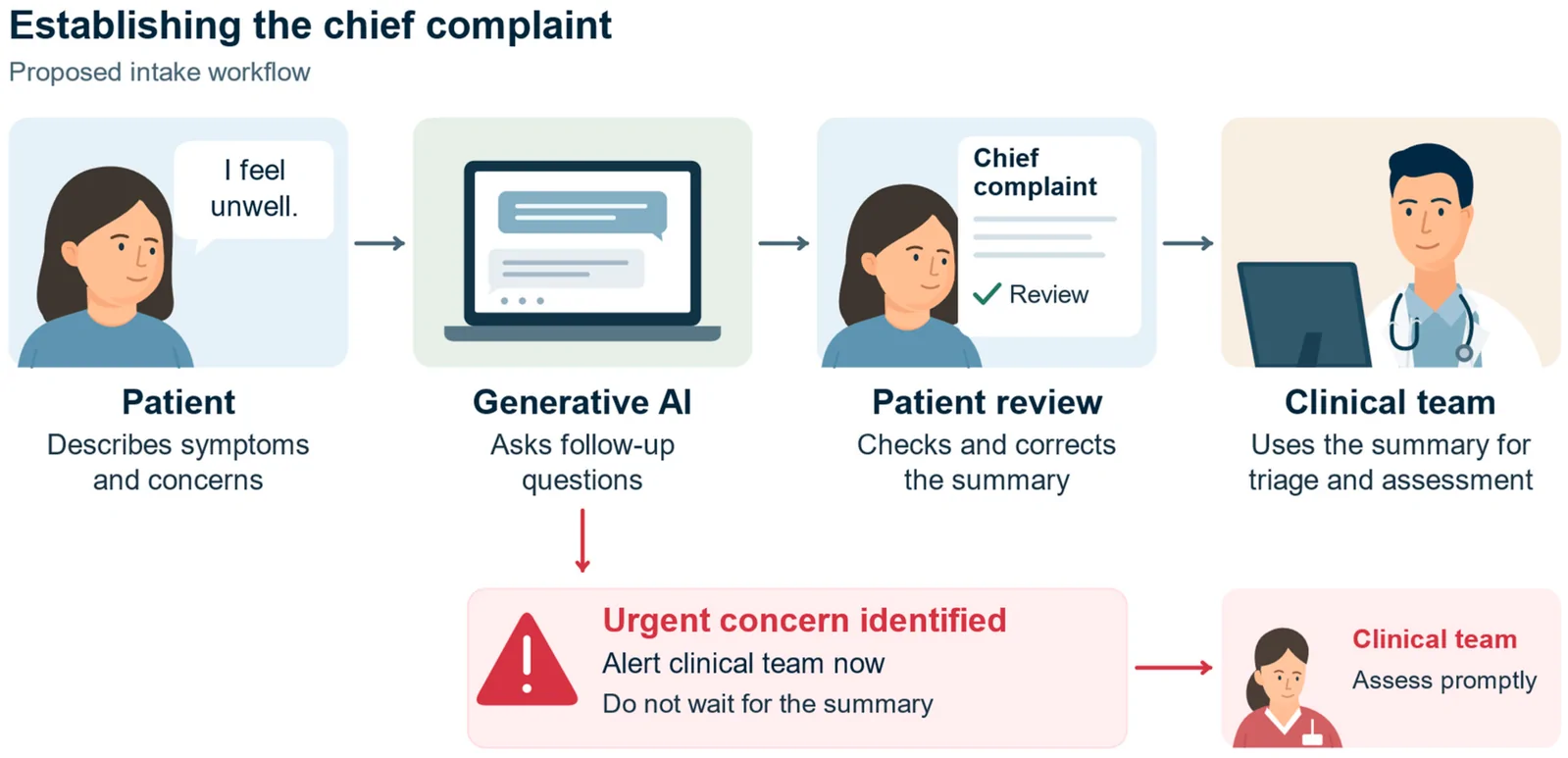
**Proposed AI-assisted intake for establishing the chief complaint.** The AI agent asks follow-up questions about the patient’s symptoms and concerns. The patient checks and corrects the summary that the clinical team uses for triage and assessment. The red pathway sends urgent concerns directly to the clinical team without waiting for the interview or patient review to finish.

Figure 1 shows how AI-assisted intake could establish the chief complaint and pass urgent concerns directly to the clinical team. Figure 1 was prepared with generative AI assistance through ChatGPT 6.0 Pro (OpenAI), then redrawn as vector artwork and checked by the authors; it illustrates a proposed workflow, not research data.

## 3. Evidence for AI-Assisted Intake

Clinical studies have begun to test whether AI-assisted questioning can prepare patients and clinicians for a consultation (Table 2). In a two-center randomized trial in China, PreA collected histories and generated referral reports before specialist visits [14]. The trial randomized 2138 participants and analyzed 2069. Physician consultation time averaged 3.14 min when patients used PreA without assistance, compared with 4.41 min without PreA; communication ratings also improved. Patients spent about 3.5 min interacting with the chatbot. The reports included suggested diagnoses and tests as well as histories, so the trial evaluated a broader intervention than chief-complaint collection alone.

The communication findings concerned the specialist visit following AI intake. Patients and care partners who used PreA without staff assistance rated ease of communication at 3.99, compared with 3.44 without PreA, on a five-point scale. They also rated the physician as more attentive. Specialists judged the AI reports more helpful for care than the control reports, which contained only age and sex. The reports were available before the specialist spoke with the patient. In end-of-shift feedback, 56 of the 72 physicians who found PreA useful or very useful identified history collection among its most valuable features [14].

A small, nonrandomized study of Spine-GPT reported more complete recorded histories on an 18-item assessment [20]. Patients attended a specialty clinic, where the clinical problem had already been narrowed.

In a prospective pediatric emergency department pilot, 31 patients or caregivers used ChatGPT-4 to provide histories while waiting [21]. Participants and physician reviewers rated the summaries favorably, although clinicians identified missing details about previous visits. Histories were collected alongside usual care and did not inform clinical decisions.

In a prospective feasibility study at Healthcare Associates, Beth Israel Deaconess Medical Center in Boston, 100 primary care patients completed supervised text conversations with AMIE before scheduled appointments [22]. Patients generally rated the experience positively, and clinicians found the output useful in preparing for the visit. This preprint describes feasibility and user experience in a selected, supervised population; it does not establish improved chief-complaint accuracy or effectiveness in routine care.

In follow-up interviews with 20 patients, participants described using AMIE to organize what they wanted to discuss before seeing the clinician. They appreciated detailed questions about their complaints and language they could understand. Ten clinicians with the most study patients were also interviewed. They described patients arriving with their thoughts and questions better organized and summaries that contained more detail than usual intake notes. The clinicians reported being able to check information already collected and then use more of the visit to explore concerns, counsel patients, and discuss management [22]. These interview findings suggest that earlier questioning may help patients bring specific questions to the consultation, even when it does not identify an additional symptom.

Two of the 114 patients who started a chat were excluded after pregnancy- or mental-health-related concerns emerged. In both cases, what the patient wanted to discuss differed from the complaint recorded in the chart [22].

Patients may need help using a chatbot. During development of the P&P Care chatbot, participants described difficulty starting a dialogue, correcting errors, and controlling how long it continued [25]. Can patients start describing their symptoms and correct a misunderstanding without special instruction? A system intended to help patients express themselves should be tested with people who are unfamiliar with it, including those who need assistance [26].

Language and cultural background can affect participation in a clinical conversation. In an observational primary care study, patient participation differed by ethnic background and was associated with clinicians’ communication [27]. In pediatric emergency visits, professional interpreters were associated with a lower proportion of interpretation errors with potential clinical consequences than informal or no interpreters [28]. AI intake should ask patients to explain unfamiliar symptom terms and provide access to trained interpreters when needed.

In a randomized crossover study of 159 simulated cases, AMIE achieved higher diagnostic accuracy and favorable ratings across several consultation and communication measures compared with primary care physicians using text chat [13]. The system had to obtain information through conversation with patient actors. However, the actors followed defined scenarios, and the comparison does not establish performance with patients trying to describe their own symptoms during routine intake.

A separate study assessed GPT-4 on 1000 supplied descriptions of symptoms and healthcare needs, using a predefined list of labels [23]. Reported performance was similar to that of a supervised classifier. The large real-world deployment described in that paper used the non-generative classifier, not GPT-4. These results do not establish whether a conversation would obtain information the patient had not initially mentioned.

The remaining question is whether clinicians can establish the chief complaint more accurately with AI assistance. Studies should test whether AI questions help patients explain unclear symptoms or disclose additional concerns. They should then examine how that information affects triage and clinical assessment.

## 4. Errors and Clinical Evaluation

Errors can occur while asking questions or while preparing the summary. A concern may remain unspoken because the AI agent never asks about it. Alternatively, the patient may state the concern clearly and the summary may omit it. These failures require different corrections. In a retrospective study of 100 emergency department notes, GPT-4 summaries omitted clinically relevant information in 47% of cases [24]. This was summarization of existing notes, not AI-assisted intake, and the percentage is not a missed-chief-complaint rate. It does show why reviewing the conversation and the summary are separate parts of evaluating an intake system.

A summary can also change the meaning of information it retains [29]. A patient’s statement, “I think this is anxiety,” could be rewritten as “Symptoms caused by anxiety.” The summary could give an exact onset date despite the patient’s uncertainty, or attribute a caregiver’s observation to the patient. Evaluation should compare these details with the actual answers. Patient review is useful, but approval of a summary does not establish that every detail is correct. Researchers need to examine whether corrections were invited, whether patients made them, and whether those corrections reached the clinician. Clinicians should also confirm important details with the patient to avoid relying on a fluent but incomplete AI summary.

The interaction also needs testing. A randomized study of 1298 members of the public found that assistance from a large language model did not improve their choice of an appropriate course of action for fictional medical scenarios compared with the control condition [30]. Some participants left out relevant information, and the models sometimes misinterpreted their questions. Participants were not patients describing their own illnesses, so this was not a clinical intake trial. Answering a fully described case does not show that an AI agent can obtain the same details in conversation.

Some benchmarks supply the chief complaint before testing the AI agent. DxDirector begins with a patient-style chief complaint generated from an existing clinical case and obtains further information step by step [31]. Such a design can test useful questioning and reasoning within the supplied problem. It cannot show whether the system would have elicited that problem from a patient with several concerns. Patient selection can also limit what the AI agent is tested on: the AMIE feasibility study enrolled patients with one chief complaint who had already been triaged away from emergency care [22]. These are limits on what the studies establish, not evidence that the systems lack the untested ability.

To test whether AI can establish the chief complaint, researchers should first ask patients separately what they hoped to discuss. Use the same neutral question in both groups, such as ‘What would you like to discuss today?’, without symptom examples, suggested priorities, or feedback. The earlier study of additional-concern questions used previsit lists to identify concerns that were not addressed [11]. The list should not be supplied to the AI agent when its ability to elicit concerns is being tested. Researchers could then compare the list with the conversation, the summary, and the subsequent clinical assessment [32]. Patients may identify further concerns during the visit, so the initial list may be incomplete [33].

Patients can report whether their main concerns were represented and whether a summary changed what they meant [16,34]. Clinicians can assess whether relevant symptom information was missing or misleading and whether a concern required earlier attention. Agreement with one phrase in the medical record is too narrow a measure of success. Several descriptions may accurately convey the same complaint. A concern explicitly deferred with a plan should also be distinguished from one that was never recognized.

Two clinicians experienced in triage should independently judge urgency using prespecified criteria appropriate to the setting, such as the 2025 Canadian Triage and Acuity Scale (CTAS) for emergency department studies [2], with access to symptoms, observations, and vital signs recorded at intake. Report percentage agreement and weighted kappa for urgency ratings before a third clinician resolves disagreements. For comparisons of AI and usual-care summaries, reviewers should receive a common format without labels identifying the source.

A primary outcome could be the proportion of patients with a main symptom or concern missing or incorrectly described in the intake summary. For patients sent directly to clinical staff before the summary is complete, reviewers should assess what information staff received at that point. Reviewers should explain why each error mattered: whether it changed the symptom being assessed, left a patient’s concern unaddressed, or removed information relevant to urgency [35]. An omitted detail that would not affect these judgments should be recorded separately. Studies should also record concerns obtained through AI questioning that were absent from the usual intake record, and count each distinct concern once per patient, combining repeated descriptions of the same symptom. The number of concerns missed and whether any main concern was missed should be reported; urgent omissions should be reported separately, without an overall severity-weighted score.

The study population should include patients with a clear single complaint, vague descriptions, and several concerns. Some should need little questioning; others may disclose a second concern only after an invitation. Simulated encounters can help examine these differences, provided the actor does not automatically volunteer every important detail. Cases should also include changes in symptoms and patient corrections, so that the test examines whether the summary is updated. Evaluations restricted to completed conversations would miss the experience of patients who stop, need help, or choose to speak directly with staff. The study should report completion, missed-concern rates, and correction rates by health literacy, preferred language, age, and access to digital devices or assistance, with subgroup sizes shown.

Studies should compare intake with and without AI assistance. The appropriate comparison may be nurse questioning, a brief form, or usual clinician intake. A brief concern form made available to clinicians at the same stage as the AI summary would help test whether AI questioning adds useful information. For triage, researchers should examine whether concerns requiring prompt review were missed and how long it took for relevant information to reach the responsible clinician. They should also count unnecessary alerts or referrals for urgent assessment. Reviewer ratings alone do not show whether a summary improves triage decisions or reduces delays [36].

Studies should measure the time patients and staff spend on intake. A shorter physician consultation can follow a longer patient interview or substantial staff assistance. Researchers should measure time to initial assessment, time answering questions, and time checking or correcting the summary. They should also record useful concerns that lead to additional assessment. Discovering a previously unmentioned problem may appropriately lengthen a visit [37]. That result should be judged by what the added assessment accomplished, rather than treated automatically as a failure to save time.

## 5. Conclusions

Generative AI could provide patients with more opportunity to describe what is troubling them and help clinicians obtain the information needed for triage and assessment. The proposed evaluation framework compares patients’ concerns with the questions asked, the summary, and the information received by clinical staff. It should show where concerns are missed or altered and whether urgent information reaches the team in time for assessment.

## Figures and Tables

**Table 1:** Clinical tasks and proposed AI assistance in establishing the chief complaint.

Clinical Task	How AI Could Assist	Information Needed By the Clinician	Proposed Measures
Clarify symptoms or concerns [3,10]	Ask patients what is troubling them and clarify vague descriptions such as “dizzy.” Ask what prompted a request for a test.	Symptoms or concerns prompting care, in the patient’s own words where needed. A suspected cause is recorded as the patient’s explanation.	Proportion of patients with a main symptom or concern missing or described incorrectly.
Establish timing and recent changes [3]	Ask when symptoms began, whether they stopped or returned, and what has changed. Ask the patient to explain conflicting dates.	When symptoms began and what changed, including any uncertainty about dates.	Proportion of summaries with an incorrect onset or recent change.
Ask about additional concerns and the patient’s priorities [11,15]	Ask what else is troubling the patient before pursuing one symptom in detail. Ask which concern they most want addressed.	Additional symptoms or concerns and the patient’s main priority, including concerns they prefer to discuss directly with a clinician.	Proportion of patients with an additional concern not elicited; report missed main concerns separately.
Provide symptom information for triage [1,2]	Notify triage staff as soon as a patient reports a potentially urgent symptom. Keep other concerns visible.	Symptoms and timing relevant to urgency, available before the interview is complete and considered alongside clinical observations and vital signs.	Proportion of urgent concerns missed; time from disclosure to staff notification; number of unnecessary alerts.
Check and update the recorded chief complaint [16]	Prepare a short summary for patients to check. Include patient corrections and newly reported symptoms in the summary sent to clinical staff.	A corrected chief complaint based on the patient’s answers, with remaining uncertainties and unanswered questions visible.	Proportion of patient corrections absent from the final summary.

References support the clinical tasks and the need to check recorded information. The AI actions are proposed uses, not demonstrated benefits for each task. Measures are proposed for clinical testing, not a validated score.

**Table 2:** Selected studies relevant to AI-assisted intake.

Study	Study Design and Setting	AI Task	Main Findings	Relevance to the Chief Complaint
Tao et al., 2026 [14] PreA	Two-center randomized trial before specialist visits; 2138 randomized and 2069 analyzed.	Collected histories and generated reports for specialists, including suggested diagnoses and tests.	Physician consultations: 3.14 min when patients used PreA without assistance versus 4.41 without PreA. Communication ratings improved.	Tests preparation for a specialist visit, beyond collecting the chief complaint. Patients also spent about 3.5 min with the chatbot.
Kaplan et al., 2026 [20] Spine-GPT	Small, nonrandomized spine-clinic pilot. *	Asked history questions in the waiting area and prepared summaries for the surgeon.	Recorded histories were more complete on an 18-item assessment.	Measures history completeness in a specialty clinic, after the clinical problem has already been narrowed.
Morley-Fletcher et al., 2026 [21]	Prospective pediatric emergency department pilot involving 31 patients or their caregivers.	Used ChatGPT-4 to collect histories and prepare summaries during the wait.	Patients and physician reviewers rated summaries favorably. Some details of previous visits were missing.	Tests history collection during emergency care. Summaries did not inform clinical decisions, so triage benefit was not tested.
Brodeur et al., 2026 [22] AMIE; preprint	Supervised primary care feasibility study at Beth Israel Deaconess Medical Center, Boston; 100 patients completed previsit conversations.	Asked patients questions through text chat and provided a transcript and summary for the clinician.	Patients generally rated the experience positively; clinicians found the output useful in preparing for the visit.	Patients had one chief complaint and had already been triaged away from emergency care before AI questioning.
Tu et al., 2025 [13] AMIE	Randomized crossover study with patient actors in 159 simulated cases.	Collected information through text conversations and proposed diagnoses.	Higher diagnostic accuracy and favorable consultation and communication ratings compared with physicians using text chat.	Tests conversation within assigned cases. The actors were not patients explaining their own symptoms during routine intake.
Naved et al., 2025 [23]	GPT-4 tested on 1000 supplied descriptions of symptoms and healthcare needs.	Assigned labels for symptoms or healthcare needs to written descriptions.	Reported classification accuracy was 0.80 for both GPT-4 and the supervised classifier.	Tests classification of concerns already expressed. The large clinical deployment used the non-generative classifier, not GPT-4.
Williams et al., 2025 [24]	Retrospective study of 100 emergency department clinician notes.	Generated encounter summaries from existing notes.	GPT-4 summaries omitted clinically relevant information in 47% of cases.	Shows information can be lost during summarization. The 47% is not a missed-chief-complaint rate during patient intake.

* The Spine-GPT report gives inconsistent sample counts; no count is reproduced here. AMIE: Articulate Medical Intelligence Explorer.

## Data Availability

No new data were created or analyzed in this study. Data sharing is not applicable to this article.

## References

[1] Chang R., Barnes D.K. (2020). Some Patients Can’t Wait: Improving Timeliness of Emergency Department Care. PSNet.

[2] Hall J.N., McCarron J., Toarta C., McLeod S.L., CTASNational Advisory Committee, NENATriage Committee (2025). Canadian Emergency Department Triage Acuity Scale (CTAS) Guidelines 2025. Can. J. Emerg. Med..

[3] Newman-Toker D.E., Cannon L.M., Stofferahn M.E., Rothman R.E., Hsieh Y.H., Zee D.S. (2007). Imprecision in patient reports of dizziness symptom quality: A cross-sectional study conducted in an acute care setting. Mayo Clin. Proc..

[4] Steitz B.D., Turer R.W., Lin C.-T., MacDonald S., Salmi L., Wright A., Lehmann C.U., Langford K., McDonald S.A., Reese T.J. (2023). Perspectives of Patients About Immediate Access to Test Results Through an Online Patient Portal. JAMA Netw. Open..

[5] Kowalski C.P., McQuillan D.B., Chawla N., Lyles C., Altschuler A., Uratsu C.S., Bayliss E.A., Heisler M., Grant R.W. (2018). “The Hand on the Doorknob”: Visit Agenda Setting by Complex Patients and Their Primary Care Physicians. J. Am. Board. Fam. Med..

[6] Beckman H.B., Frankel R.M. (1984). The effect of physician behavior on the collection of data. Ann. Intern. Med..

[7] Ospina N.S., Phillips K.A., Rodriguez-Gutierrez R., Castaneda-Guarderas A., Gionfriddo M.R., Branda M.E., Montori V.M. (2019). Eliciting the Patient’s Agenda—Secondary Analysis of Recorded Clinical Encounters. J. Gen. Intern. Med..

[8] Barry C.A., Bradley C.P., Britten N., Stevenson F.A., Barber N. (2000). Patients’ unvoiced agendas in general practice consultations: Qualitative study. BMJ.

[9] Allgood S., Park J., Soleiman K., Saha S., Han D., McArthur A., Moore R.D., Beach M.C. (2023). Taxonomy and effectiveness of clinician agenda-setting questions in routine ambulatory encounters: A mixed method study. Patient Educ. Couns..

[10] Heritage J., Robinson J.D. (2006). The structure of patients’ presenting concerns: Physicians’ opening questions. Health Commun..

[11] Heritage J., Robinson J.D., Elliott M.N., Beckett M., Wilkes M. (2007). Reducing patients’ unmet concerns in primary care: The difference one word can make. J. Gen. Intern. Med..

[12] Rueegg M., Nickel C.H., Bingisser R. (2021). Disagreements between emergency patients and physicians regarding chief complaint—Patient factors and prognostic implications. Int. J. Clin. Pract..

[13] Tu T., Schaekermann M., Palepu A., Saab K., Freyberg J., Tanno R., Wang A., Li B., Amin M., Cheng Y. (2025). Towards conversational diagnostic artificial intelligence. Nature.

[14] Tao X., Zhou S., Ding K., Li S., Li Y., Wu B., Huang Q., Chen W., Shen M., Meng E. (2026). An LLM chatbot to facilitate primary-to-specialist care transitions: A randomized controlled trial. Nat. Med..

[15] Brock D.M., Mauksch L.B., Witteborn S., Hummel J., Nagasawa P., Robins L.S. (2011). Effectiveness of intensive physician training in upfront agenda setting. J. Gen. Intern. Med..

[16] Bell S.K., Delbanco T., Elmore J.G., Fitzgerald P.S., Fossa A., Harcourt K., Leveille S.G., Payne T.H., Stametz R.A., Walker J. (2020). Frequency and Types of Patient-Reported Errors in Electronic Health Record Ambulatory Care Notes. JAMA Netw. Open..

[17] Van Veen D., Van Uden C., Blankemeier L., Delbrouck J.-B., Aali A., Bluethgen C., Pareek A., Polacin M., Reis E.P., Seehofnerová A. (2024). Adapted large language models can outperform medical experts in clinical text summarization. Nat. Med..

[18] Wang M.D., Khanna R., Najafi N. (2017). Characterizing the Source of Text in Electronic Health Record Progress Notes. JAMA Intern. Med..

[19] Anderson M.O., Jackson S.L., Oster N.V., Peacock S., Walker J.D., Chen G.Y., Elmore J.G. (2017). Patients Typing Their Own Visit Agendas Into an Electronic Medical Record: Pilot in a Safety-Net Clinic. Ann. Fam. Med..

[20] Kaplan A., Kaplan B.A., Aydilek A., Ege T., Şentürk S., Solmaz I. (2026). Spine patient history-taking: A two phase study of a prompt-engineered large language model assistant. Eur. Spine J..

[21] Morley-Fletcher A., Raghavan V.R., Geanacopoulos A.T., Maher M., Waltzman M., Corren Y.B., Fine A.M. (2026). A Pilot Study to Evaluate Artificial Intelligence-Driven Early Retrieval of Medical Histories in the Emergency Department. Ann. Emerg. Med..

[22] Brodeur P., Koshy J.M., Palepu A., Saab K., Homiar A., Ruparel R., Wu C., Tanno R., Xu J., Wang A. (2026). A prospective clinical feasibility study of a conversational diagnostic AI in an ambulatory primary care clinic. arXiv.

[23] Naved B.A., Ravishankar S., Colbert G.E., Johnston A., Slott Q.M., Luo Y. (2025). LLM enabled classification of patient self-reported symptoms and needs in health systems across the USA. npj Digit. Med..

[24] Williams C.Y.K., Bains J., Tang T., Patel K., Lucas A.N., Chen F., Miao B.Y., Butte A.J., Kornblith A.E. (2025). Evaluating large language models for drafting emergency department encounter summaries. PLoS Digit. Health.

[25] Li S., Li Y., Zhou S., Tao X., Yu C., Shen M., Chen W., Meng E., Wu B., Huang Q. (2026). A community-codesigned LLM-powered chatbot for primary care: A randomized controlled trial. Nat. Health.

[26] Bickmore T.W., Pfeifer L.M., Byron D., Forsythe S., Henault L.E., Jack B.W., Silliman R., Paasche-Orlow M.K. (2010). Usability of conversational agents by patients with inadequate health literacy: Evidence from two clinical trials. J. Health Commun..

[27] Schouten B.C., Meeuwesen L., Tromp F., Harmsen H.A.M. (2007). Cultural diversity in patient participation: The influence of patients’ characteristics and doctors’ communicative behaviour. Patient Educ. Couns..

[28] Flores G., Abreu M., Barone C.P., Bachur R., Lin H. (2012). Errors of medical interpretation and their potential clinical consequences: A comparison of professional versus ad hoc versus no interpreters. Ann. Emerg. Med..

[29] Asgari E., Montaña-Brown N., Dubois M., Khalil S., Balloch J., Yeung J.A., Pimenta D. (2025). A framework to assess clinical safety and hallucination rates of LLMs for medical text summarisation. npj Digit. Med..

[30] Bean A.M., Payne R.E., Parsons G., Kirk H.R., Ciro J., Mosquera-Gómez R., Hincapié M.S., Ekanayaka A.S., Tarassenko L., Rocher L. (2026). Reliability of LLMs as medical assistants for the general public: A randomized preregistered study. Nat. Med..

[31] Xu S., Huang X., Wei Z., Pang L., Shen H., Cheng X. (2026). DxDirector: An agentic large language model driving the full-process clinical diagnosis. Nat. Commun..

[32] Bell S.K., Dong Z.J., Desroches C.M., Hart N., Liu S., Mahon B., Ngo L.H., Thomas E.J., Bourgeois F. (2023). Partnering with patients and families living with chronic conditions to coproduce diagnostic safety through OurDX: A previsit online engagement tool. J. Am. Med. Inform. Assoc..

[33] Peltenburg M., Fischer J.E., Bahrs O., van Dulmen S., van den Brink-Muinen A. (2004). The unexpected in primary care: A multicenter study on the emergence of unvoiced patient agenda. Ann. Fam. Med..

[34] Early F., Everden A.J.T., O’Brien C.M., Fagan P.L., Fuld J.P. (2015). Patient agenda setting in respiratory outpatients: A randomized controlled trial. Chron. Respir. Dis..

[35] Liu X., Rivera S.C., Moher D., Calvert M.J., Denniston A.K., Ashrafian H., Beam A.L., Chan A.-W., Collins G.S., Darzi A. (2020). Reporting guidelines for clinical trial reports for interventions involving artificial intelligence: The CONSORT-AIExtension. BMJ.

[36] Vasey B., Nagendran M., Campbell B., A Clifton D., Collins G.S., Denaxas S., Denniston A.K., Faes L., Geerts B., Ibrahim M. (2022). Reporting guideline for the early stage clinical evaluation of decision support systems driven by artificial intelligence: DECIDE-AI. BMJ.

[37] Middleton J.F., McKinley R.K., Gillies C.L. (2006). Effect of patient completed agenda forms and doctors’ education about the agenda on the outcome of consultations: Randomised controlled trial. BMJ.

